# Emotional Effects of Live and Recorded Music in Various Audiences and Listening Situations

**DOI:** 10.3390/medicines6010016

**Published:** 2019-01-22

**Authors:** Töres Theorell, Eva Bojner Horwitz

**Affiliations:** 1 Department of Music, Pedagogy and Society, Royal College of Music, Box 27711, SE-115 91 Stockholm, Sweden; tores.theorell@ki.se; 2 Department of Clinical Neuroscience, Karolinska Institutet, SE-171 77 Stockholm, Sweden

**Keywords:** age, anxiety, arousal, joy, live music, recorded music

## Abstract

**Background:** We assume that the emotional response to music would correspond to increased levels of arousal, and that the valence of the music exemplified by sad or joyful music would be reflected in the listener, and that calming music would reduce anxiety. This study attempts to characterize the emotional responses to different kinds of listening. **Methods:** Three experiments were conducted: (1) School children were exposed to live chamber music, (2) two adult audiences who were accustomed to classical music as a genre listened to chamber music, and (3) elderly listeners were exposed to recorded classical music of a sad character with and without words. Participants were asked to fill in visual analogue 10-cm scales along dimensions of: tiredness-arousal, sadness-joy, and anxiety-calmness. Ratings before exposure were compared with ratings after exposure. **Results:** The strongest positive emotional responses were observed in the live performances for listeners accustomed to classical music. School children tended to become tired during the concert, particularly the youngest children. There was a calming effect among school children, but in the oldest category increased joy was reported. **Conclusions:** The findings indicate that emotional response to music varies by type of audience (young, old, experience of classical music), and live or recorded music.

## 1. Introduction

That music listening can have strong emotional effects is widely accepted, and it is also known that the effects depend on several individual and environmental factors. There are several elements of the musical experience that influence the emotional response of the listener, of which we focus on four that have been explored in the scientific literature. Firstly, the type of music that refers to the structural features of the music (for instance, music that is assumed to be calming or stimulative) is one factor that may obviously be of importance. Secondly, individual differences—even when subjects choose their preferred type of music with its specified characteristics (stimulating or calming respectively) the emotional responses elicited may vary considerably between individuals according to, for example, personality and the social or cultural context in which they reside [1]. Despite that fact, overall, it has been noted that stimulative self-selected music tends to have a strong association with joy and energy, and the calming music in general has an anxiety reducing effect. Thirdly, whether an individual listens to recorded or live music, it has been shown to be of significance for the emotional response to music [2]. The fourth and final factor we wish to highlight is listener familiarity with the piece of music that they listen to—if there are strong prior emotional experiences attached to a specific piece of music, the emotional effects may be amplified as compared to those experienced by other people. For instance, some individuals may respond with strong anxiety to a piece of music regarded as calming by most listeners and react with joy to a piece regarded as sad by other people. There is extensive literature on these factors, as summarized for instance by Juslin and Sloboda [3]. The patterns of emotional reactions during music listening are admittedly very complex, as discussed by Salimpoor et al. [4]. Responses related to arousal are related to basic elements of the music, such as volume, timbre, pitch, and tempo [5]. Unexpected elements in the music may give rise to arousal as well as to joyful, sad, or worried reactions if the emotional message is in conflict with the emotional state that the person happens to be in when the listening starts [6].

The aim of the present study is to assess emotional responses to music across three age groups, drawing on contrasting music listening situations. The design of the study mobilises a simple paradigm mainly based on James Russell’s elaborated version of his original circumplex model of affect [7], which has been used quite extensively in the music and emotional literature by Schubert [8], Eerola and Vuoskoski [9], and Schimmack and Grob [10], among others. Russell’s model is composed of two axes: valence (unpleasant to pleasant) and arousal (deactivation to activation), and its framework describes how the affective concepts of pleasure, excitement, arousal, stressful, displeasure, depression, sleepiness, and relaxation fit into this model. This model of emotional axis thinking has also been linked in further work to physiological theory [11]. 

Although many experiments have been performed in the past on emotional effects of music listening, no efforts have been made in the direction of standardizing a simple measurement tool for the most crucial emotional reactions. In the present study we test such a tool in contrasting situations—in the class room versus in the lecture or concert room and with widely different audiences in different ages ranging from school children to elderly subjects—and with large differences in experience with the kind of music that they were exposed to. The only factor that was kept constant throughout the experiments was genre. Only classical music was played. We predict that emotional responses to the musical pieces will be widespread across age groups, degree of musical background, familiarity with specific pieces of music, and listening conditions (live or recorded).

## 2. Materials and Methods 

The emotional assessment tool that we have devised and tested has three dimensions, namely, arousal, joy, and calmness. The two first dimensions correspond to the axes in the circumplex model and the third dimension, worry-calmness, is an addition. The third dimension was chosen because it is particularly relevant in the study of reactions to music among young subjects [12]. 

**Figure 1:**
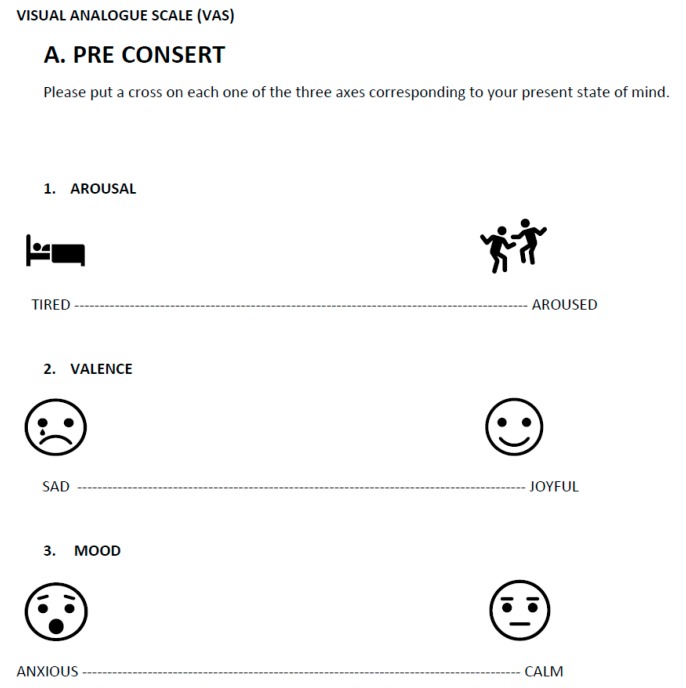
Presents the Visual Analog Scale (VAS) used before (PRE) and after (POST) all the exposures.

Three 1-dm horizontal lines were used with the extremes tired (left) to alert (right), sad (left) to happy (right), and worried (left) to calm (right), respectively. The adjectives were chosen after pilot tests with a larger selection of words (see Figure 1).

The Visual Analogue Scales (VAS) methodology selected has gone through two validity test studies in music listening situations [1,2]. The first test was performed when young adults listened to one “stimulating” and one “relaxing” recorded piece of music, both of them self-selected favorites. According to the VAS recordings, the “stimulating” music gave rise to a strong arousal reaction while the “relaxing” music was associated with a significant decrease in energy. Both favorites stimulated joy and calmness significantly. The second test [2] was performed on employees who participated in a worksite-based culture experiment during work hours, with cultural experiences offered once every week for three months. These validity tests provide evidence that the VAS scales do reflect the three emotional states that are our focus in the present study and that experimental subjects are able to discriminate between the feelings. 

The VAS methodology has been subjected to extensive psychometric testing. Visual Analogue Scales and similar paper and pencil tests have a strong tradition of use in psychometric practice, being widely used in pain measurement [12] and also in assessments of physical effort [13] and fatigue [14]. 

Our test sought the answer to the following questions: Does the technique adequately mirror expected differential emotional reactions in the three dimensions? Do we find differences in responses between listeners who differ with regard to age, familiarity with the pieces of music, and when the music is recorded versus live?

The questionnaire was designed to be simple to administer immediately in connection with the listening experiences. It should be easy for participants to fill in the sheets before and after the stimulus (see Figure 1).

### 2.1. Study Groups (See Table 1)

#### 2.1.1. Students at School Listening to Live Chamber Music

Ulriksdal’s School is a public school. Children in the third and fifth grades (23 children in each class) were invited to listen to live chamber music played by a trio (violin, viola, and cello) consisting of one teacher and two advanced students in chamber music. There was no introductory lecture about the music. Separate performances were made in each of the two classrooms. After a solo performance by the viola player (excerpt of a partita by Johann Sebastian Bach) the ensemble played Beethoven’s string trio, in G-major, op 9 number 1, as well as the Serenade for string trio by Ernst Dochnanyi op 10. These latter two pieces are full of contrasts and are regarded by most people as arousing music. The duration of the chamber music listening was nearly 30 min.

The Kristoffer School is a Waldorf school. The pupils in grade 7 and 8 (20 in each class) were invited to listen to a concert in the class room with separate but identical programs in the two class rooms. The musicians who were all advanced students in chamber music formed a string quartet (two violins, a viola, and a cello). After an introductory partita for violin by JS Bach, they played Mozart’s string quartet number 15 (K nr 458) in B flat major, which is a joyful and lively piece of music. The duration of the chamber music listening was nearly 30 min.

#### 2.1.2. Experienced Chamber Music Listeners in Two Concerts

In the first concert, the audience was invited to this special occasion (inauguration of the Musethica program in Stockholm), and in the second case the audience was participating in the inauguration of the new building for the Royal College of Music. This latter chamber music concert was one of many concerts offered as part of a free program that day. The performing trio was the same as in the Ulriksdal’s School. The Beethoven trio and the Dochnanyi serenade were performed as previously. 

#### 2.1.3. Elderly Persons Listening to a Lecture at the “Senior University” in Stockholm

There were 110 participants aged 63 to 85 in the lecture. However, due to technical problems, only 92 subjects could participate in the first session. The lecture was divided in two parts with an intermission between. During both sessions the listening with visual analogue scales (VAS) scoring took place towards the end. During the first lecture participants listened to the beginning of a recording of the second movement of Franz Schubert’s string quintet in C minor, which is a sad piece of music. During the second lecture they listened to two of the last songs in Schubert’s singing cycle Winterreise (Die Krähe and Der Leiermann). The lecturer leading the sessions was the amateur musician who performed in both recordings. The duration of the music piece in both cases was seven minutes.

Questionnaires were distributed to all the participants in the audience before the listening session began. The scales for recording pre-listening were displayed on the front page. The listeners were instructed to put a cross on each one of the three axes corresponding to their present state of mind. After listening, they were asked to turn the page where the post listening scales were printed. They were instructed to fill out the post listening scores without taking the pre-listening scores into account (Figure 1).

Table 1 shows the demographic characteristics of the study samples. The different samples have significantly different age spans. Among the school children there is an almost equal share of boys and girls. In the older samples there is a dominance of women. As expected, the youngest children reported very little previous experience of listening to or performing classical music. The musethica sample has the highest degree of experience of classical music, while the music academy listeners and the elderly subjects report a dominance of a medium experience. The vast majority of listeners to both the concerts and to the recorded music during the lecture had university education. 

Non-participation was null in the children and below five percent in the other samples. The n is lower for the first occasion in the experiment with the elderly. This is due to technical difficulties with the sound amplification in the lecture hall on the first listening occasion - the sound was inaudible in part of the room.

### 2.2. Statistical Methods

Both means (with standard deviations) and medians (with maxima and minima) were calculated both before and after listening for all the emotional states. Significance of changes pre-post in reported emotional states was computed by means of two-tailed Wilcoxon tests. In the school sample, a multiple linear regression analysis was performed with the explanatory variables age, gender, and music experience, and change in arousal level as dependent variable. Non-parametric tests were used as the scales have an ordinal character, although they are close to being normally distributed. Since the distributions were close to normal distributions, we considered it appropriate to use multiple linear regression to test a more limited question regarding the children: Do the variables “age”, “previous experiences”, and “gender” have mutually independent values in the prediction of emotion change?

### 2.3. Ethical Approval

Ethical approval was obtained for the study (Dnr 2017/1009-31/1 Central Ethical Review Board in Stockholm, Sweden), and both oral and written informed consent was obtained from the participants.

## 3. Results

**Figure 2:**
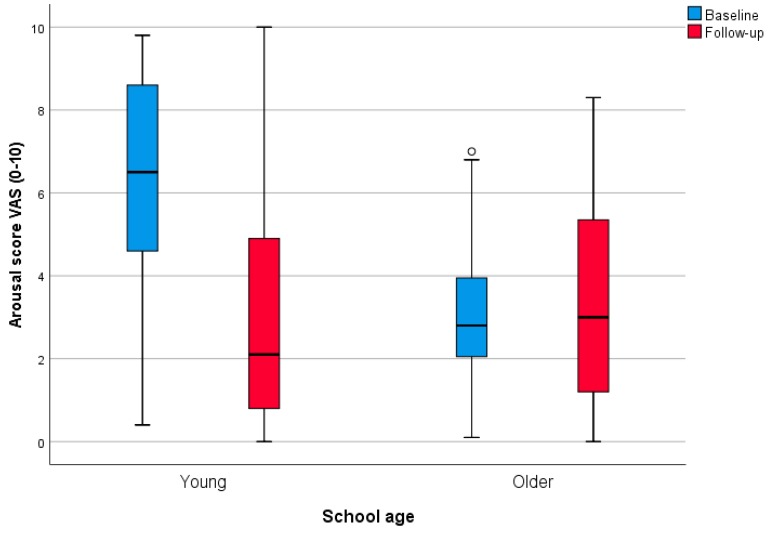
Boxplot (Median, minimum-maximum) for arsousal scoring in youth: Younger = Grade 3 and 4; Older = Grade 7 and 8. Scores are assessed using VAS with range 0–10. The small circle represents one outlier.

Figure 2 shows the results from the VAS ratings in the children before and after concert, grouped into two categories: listeners in grades 3 and 4, and listeners in grades 7 and 8. It is observed that arousal decreased significantly (−3.3) in the younger children (*p* < 0.001), but the change was less clear in the older category (*p* = 0.211). With regard to valence, there was an increase in joy in the older children (+0.5) (*p* = 0.045) but no such change in the younger ones (*p* = 0.733). Calmness increased in the younger children (+0.7) (*p* = 0.045), and was also observed although to a smaller degree in the older category (+0.3) (*p* = 0.052).

**Figure 3:**
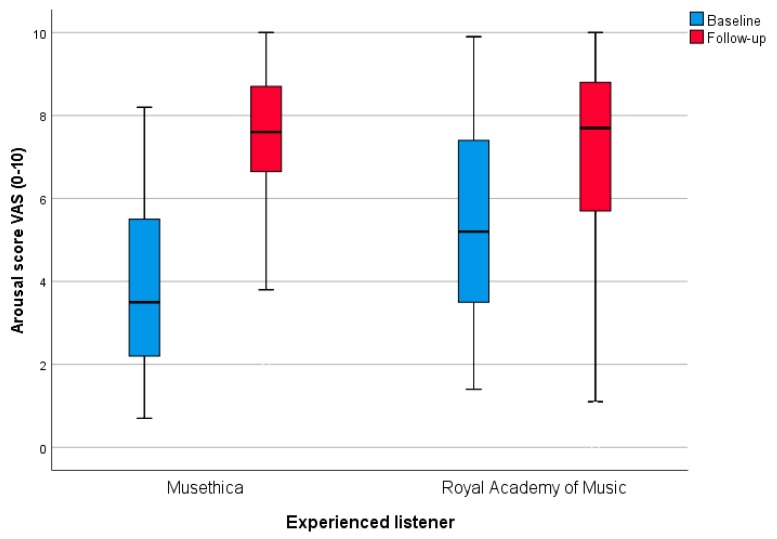
Boxplot (median, minimum-maximum) for arsousal scoring in experienced listeners—Musethica, Royal Academy of Music. Scores are assessed using VAS with a range of 0–10.

Figure 3 shows the results from the VAS ratings from the audiences that are accustomed to the genre of classical music but not necessarily experts of classical music, before and after the concerts. 

There were pronounced changes in VAS ratings on both occasions, and all of them were significant. On the first occasion, the change in mean arousal rating was +4.6 (*p* = 0.001), and on the second occasion it was +2.2 (*p* < 0.001). Correspondingly, the increase in joy rating was +1.0 (*p* = 0.003) and +2.0 (*p* < 0.001), respectively. The calmness rating increased +2.0 (*p* = 0.001) and +1.4 (*p* < 0.001), respectively. See Table 2.

**Figure 4:**
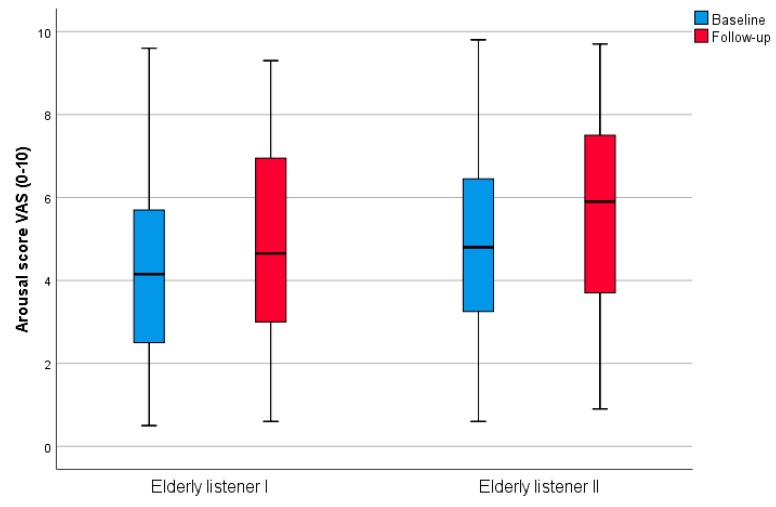
Boxplot (median, minimum-maximum) for arsousal scoring in subjects from the senior university. Scores are assessed using VAS with a range of 0–10.

Figure 4 shows the results from listening to recorded music. The audience from the “senior university” in Stockholm was relatively old and accustomed to the genre of classical music. The context was a session of listening to recorded music during a lecture about music perception. Listening was confined to two short 7-min-periods, one during the latter part of the first half of the lecture (n = 91) and one during the latter part of the second part (n = 111), with an intermission between sessions. In contrast to the other experiments, the atmosphere of both recorded pieces was sad, the first piece being the introductory part of the second movement in Schubert’s string quintet (which is frequently used as funeral music for elderly people), and the second piece being two sad songs from Schubert’s Winterreise (Die Krähe and Der Leiermann).

After the first listening occasion there tended to be a weak and non-significant arousal effect (+0.4) (*p* = 0.113) and a significant sadness effect (−0.9) (*p* < 0.001). In addition, the participants reported a significantly decreased calmness (−1.1) (*p* = 0.001) effect of the listening. After the second occasion there was a significant (and stronger) arousal effect (+0.7) (*p* < 0.001) as well as a weak sadness effect indicated (−0.4) (*p* = 0.093). There was no significant change in calmness (*p* = 0.343) after the second listening occasion. See Table 2.

The arousal scores before music listening differed significantly between the groups. The 95% confidence interval for the younger group of children (5.5–7.1, mean 6.3) was above the arousal means for all the other groups and even above the corresponding confidence intervals of all other groups, with the exception of the listeners in the music academy (4.6–6.0), although the difference was in the same direction for that group as well. On the other hand, the confidence interval (2.3–3.5, mean 2.9) for the arousal mean in the group of older children was below the confidence intervals for all other groups, with the exception of the Musethica audience (3.0–4.8), although the mean for the older children was also below that mean. Thus, the younger children had a remarkably high arousal mean, while the older teen-aged children had a remarkably low mean compared to the other groups. For joy and calmness, there were no significant starting differences between the groups. See Table 2.

## 4. Discussion

This investigation confirmed that samples of individuals from widely different age groups, who had different preferences for music and different backgrounds with regard to music, are affected to different degrees emotionally while listening to music under different conditions. We found that arousal decreased and calmness increased to a greater extent among younger as compared to older children, whilst joy increased only in the older children. Among audiences who were accustomed to classical music, on both listening occasions, changes in the VAS ratings were pronounced, showing an increase in arousal, joy, and calmness. In testing the listening conditions of music (in this test, recorded music), the older participants who were accustomed to the classical genre had relatively weak arousal responses to the music, with a non-significant effect on the first listening occasion (significant on the second). Although sadness increased and calmness decreased significantly in the first listening, the reactions were weaker on the second occasion. 

Although our results are preliminary, the strongest positive effect that we have observed among all of our listening experiments, and even including previous listening experiments, is the arousal effect of the Musethica inauguration concert. The effects of the concert at the Royal Music Academy were also strong, but were not of the same magnitude with regard to arousal. An important background factor that could explain the strong effect observed is that the Musethica inauguration concert was a very high quality live performance with an audience that was accustomed to the kind of classical music that was played and who also had high positive expectations. The concert room was relatively small, which means that there was a more private atmosphere than in the Royal Music College concert. Moreover, the two pieces of music listened to have a similar character and mostly had a joyful stimulative character. 

With regard to type of music, it should be noted that in the present study, all listening experiences were conducted using classical chamber music. The older audiences were accustomed to this genre of music, whereas the school children were only accustomed to a limited extent. The sad and slow character of the recorded music played in the lecture situation did result in aroused, sad, and worried reactions (albeit relatively weak) as expected. Interestingly, the song (sad songs from the late part of Schubert’s Winterreise) resulted in a lesser degree of sadness and worry than the instrumental piece (slow movement of Schubert’s string quintet), which is commonly used as funeral music. One listener even made the remark that the words in the song made her slightly irritated—this may be associated with the theme of the song: a crow that pesters and reminds of coming death—“please leave me”. A Swedish translation of the German words was used so that listeners without knowledge of German could understand the words. Apart from the fact that this audience listened to recorded rather than live music, the duration of the listening was shorter, in both cases seven minutes.

Our results should also be placed in the context of other studies that we have performed with similar methods. In Lingham’s and Theorell’s study [1], young adults listened to recorded pieces of stimulative and subsequently relaxing music, which participants identified as their favorites. This was associated with an increase in arousal level of +2.8 in the former case and a decrease of -0.6 in the latter. The corresponding joy effect in that study was +2.5 for the stimulative and +0.6 for the calming piece. Accordingly, familiarity with the music (and even favorite status) is important and could make even recorded music strongly arousing and joyful. That chamber music can have an arousal effect also with adult audiences that are unaccustomed to this genre of music was shown in a previous experiment [2], which showed a significant arousal effect of +1.6 when unselected employees listened to live chamber music at their places of work. 

The experiment with the school children showed that there are strong effects of age on arousal levels—the youngest children started with a much higher arousal level and the older children with a lower arousal level than the other listeners in the study. This may very well reflect the well-known developmental differences in energy level between teenagers (in this case 13–15 years old) and younger children (in this case 9–12). A separate analysis of the two age groups showed that the decrease in arousal level was highly significant (*p* < 0.0001) in the youngest group, and non-significant in the older group. 

Most of the adult listeners were familiar with the pieces of classical music and there was no effect of degree of acquaintance (variation mainly between medium and high). Similarly, there was no age or gender effect in the adult samples. However, it was only in the children with no previous experience of the genre that the arousal level decreased significantly (*p* < 0.0001). No such effect was observed among children with such experience. This effect of previous experience was also significant (*p* = 0.008) when the age effect (*p* = 0.0003) was adjusted for in multiple linear regression. 

With regards to implications for practice, individual choice of music is an important factor. None of our musical experiments were linked to a health care context, but we have seen from previous research that soothing music can have both psychological and physiological effects, for instance on patients when listening to their own choice of music after open-heart surgery [15]. The importance of choosing own music in situations when in pain or in a state of anxiety has also been a demonstrated part of the “Star Music Project” introduced in Danish psychiatric care [16]. The project aims to examine how the self-selection process of music affects and empowers patients in painful health care situations. As a result, special software and hardware has been developed to make the process of choosing the patient’s preferred music genre as easy as possible. In another area of research, the establishment of “new sound/music milieus”, so called “sound-scapes”, was introduced by the Canadian composer Murray Schafer [17], who concluded that the human heartbeat had a strong influence and impact on how we choose music in a specific moment. Researchers have since then discussed the relationship between music stimuli and emotional response within the listener and also discussed different mechanisms in the listener that could mediate different responses [18]. Furthermore, Musical expectancy [6], visual imagery [19], evaluative conditioning [20], episodic memory [21], and contagion have been related to the internal feedback of the perceived musical experience [22]. Additionally, brain stem reflexes [23] are mechanisms that have been discussed as having potential influence on emotional responses.

As seen in our experiments, the strongest positive reactions were observed when listening to live music as opposed to a recorded piece. This is also seen in other studies, specifically, in elderly adults and in patients with dementia [24,25]. Live music in health care, as part of initiatives such as “musicians in residence”, are not yet common in our hospitals. However, preparatory programs for musicians, to help them to be prepared for work in hospitals, are in use in some university cities today (see Georgetown University).

Whether our participants were familiar with the music or not was not evaluated in this study. Further subjective information could be important indicators when systematizing live music into different health care situations, such as recognizable music, lyrics, different instruments being played, and cognition levels. Interestingly, it has been observed in other studies that people with different cognitive capacities and functional levels may respond differently to music [24]. Of course the effect of the intervention may also be correlated to baseline levels before listening to music. In this study there were substantial differences between young children and other participants with regard to arousal mean scores at baseline, with 6.3 for young children compared to levels varying from 2.9 to 5.4 in others. What the interpretation of these results should be is not clear and needs further exploration in future studies. 

The VAS technique mirrored differential emotional reactions in the three dimensions and the emotional reactions regarding live and recorded music also varied in the groups. Questions regarding, for example, how musical soundscapes affect us emotionally in our society and what that will do with our behaviors and cognitions are important questions for researchers. The continuous input and triggers from different musical genres, also other than classical, may affect us on deeper emotional levels, which could play a role when discussing, for example, benevolence and compassion in our future landscapes.

The value of this paper relates to findings of others that strong musical experiences have the capacity to significantly affect the lives of people across the entire lifespan [26], also with the added value that our findings relate to different age categories.

Our data show that the introduced questionnaire seems to be sensitive to listening experiences, even when the music is recorded and of short duration, and that it is specifically sensitive to sad themes in the music and that it mirrors differences in reaction patterns among school children depending on age and familiarity with the pieces of music. Since the instrument is easy to distribute, it should be possible to use it in music therapy sessions, both for children and for adults. An important contribution of this article is that the evaluation method could be applied in settings where “quick-checks” are useful—for example, schools and healthcare contexts.

## Figures and Tables

**Table 1:** Descriptive statistics for demographics; age, gender, and education, and subject characteristics; experience with music, by Study Group.

	Outcome Variable		Age		Gender		Education			Experience with Music			
					Male	Female	Primary	Secondary	University	No	Little	Medium	High
Category		n	Mean (SD)	Median (min-max)	n	n	n	n	n	n	n	n	n
Children	Younger	46	10.4 (1.2)	11 (9–12)	25	21	46	0	0	11	16	14	4
	Older	40	13.9 (0.6)	14 (13–15)	28	12	40	0	0	0	7	27	6
Experienced	Musethica	23	44.8 (15.5)	36 (22–70)	9	13	0	0	22	0	2	6	14
	Music Academy	66	57.6 (19.2)	65 (13–83)	23	37	4	4	48	0	13	27	19
Elderly	Elderly listener I	93	73.0 (5.1)	73 (63–85)	18	71	2	8	75	0	24	48	21
	Elderly listener II	111	72.9 (4.9)	73 (63–84)	18	90	2	9	86	0	32	49	26

**Table 2:** Descriptive statistics and results for Wilcoxon Signed Rank Test for scores in arousal, joy, and calmness by age group and music experience.

				Arousal			Joy			Calmness		
			n	Mean (SD)	Med (Min-Max)	*p*	Mean (SD)	Med (Min-Max)	*p*	Mean (SD)	Med (Min-Max)	*p*
Children	Younger	pre	46	6.3 (2.6)	6.5 (0.4–9.8)		7.3 (2.3)	8.0 (1.0–10.0)		7.6 (2.3)	8.5 (2.0–9.9)	
		post	46	3.1 (2.8)	2.1 (0.0–10.0)	<0.001	7.4 (2.5)	8.1 (1.0–10.0)	0.733	8.3 (1.9)	9.4 (1.8–10.0)	0.045
	Older	pre	40	2.9 (1.7)	2.8 (1.0–7.0)		6.0 (1.9)	6.1 (1.5–9.4)		6.5 (1.8)	7.0 (1.9–9.2)	
		post	40	3.3 (2.5)	3.0 (0.0–8.3)	0.211	6.5 (1.9)	6.8 (2.0–9.2)	0.045	6.8 (2.1)	7.5 (0.3–9.2)	0.052
Experienced	Musethica	pre	23	3.9 (2.2)	3.5 (0.7–8.2)		7.3 (2.4)	8 (1.7–10.0)		5.7 (2.4)	6.4 (1.2–9.5)	
		post	23	7.1 (2.5)	7.6 (0.7–10.0)	0.113	8.6 (1.1)	9 (5.3–10.0)	<0.001	8.3 (1.4)	8.4 (3.0–10.0)	0.001
	Music Academy	pre	66	5.4 (2.4)	5.2 (1.4–9.9)		6.8 (2.1)	7 (1.3–9.8)		6.7 (2.3)	7.1 (0.0–10.0)	
		post	63	6.8 (2.9)	7.7 (0.0–10.0)	<0.001	8 (1.8)	8.6 (1.6–10.0)	0.093	8 (1.9)	8.7 (0.0–10.0)	0.343
Elderly	Elderly listener I	pre	93	4.3 (2.4)	4.1 (0.5–9.6)		6.2 (2.0)	6.3 (1.0–10.0)		7.1 (2.2)	7.5 (1.0–10.0)	
		post	92	4.7 (2.3)	4.7 (0.6–9.3)	0.001	5.2 (2.3)	5.3 (1.0–9.4)	0.003	6 (2.4)	6.2 (1.2–10.0)	0.001
	Elderly listener II	pre	111	4.9 (2.2)	4.8 (0.6–9.8)		6 (1.9)	5.9 (1.2–9.4)		6.5 (2.2)	6.9 (1.0–9.8)	
		post	112	5.6 (2.3)	5.9 (0.9–9.7)	<0.001	5.6 (2.1)	5.6 (0.7–9.2)	<0.001	6.2 (2.4)	6.5 (0.0–9.7)	<0.001

SD = Standard Deviation, Med = Median.
